# Thyroid nodular disease after radiotherapy to the neck for childhood Hodgkin's disease

**DOI:** 10.1038/sj.bjc.6690425

**Published:** 1999-05

**Authors:** E A Shafford, J E Kingston, J C Healy, J A W Webb, P N Plowman, R H Reznek

**Affiliations:** Departments of Paediatric Oncology, Diagnostic Imaging, and Radiotherapy, St Bartholomew's Hospital, London, UK

**Keywords:** childhood Hodgkin's, thyroid disease, neck irradiation

## Abstract

Patients who receive radiotherapy to the neck are at risk of developing thyroid dysfunction. This prospective study of patients whose treatment for Hodgkin's disease in childhood included radiotherapy to the neck aimed to investigate the incidence and natural history of thyroid dysfunction and the morphological changes of the gland demonstrated on ultrasound. Forty-seven patients were investigated by clinical examination, thyroid function tests and thyroid ultrasound. Only six patients had a clinically detectable abnormality, but 64% had abnormal thyroid function tests. All patients had an abnormal thyroid ultrasound scan and 42% had at least one focal abnormality. A significant association was found between the presence of a focal lesion on ultrasound and young age at radiotherapy, longer follow-up and the length of time that the thyroid-stimulating hormone (TSH) level had been elevated. During follow-up, 65% of patients not on thyroxine developed new focal abnormalities. The longest time interval between radiotherapy and an increase in TSH level was 94 months, and from radiotherapy to the appearance of a focal abnormality on thyroid ultrasound was over 18 years. Three patients were found to have a thyroid carcinoma. These findings indicate the importance of long-term follow-up for patients treated by neck irradiation for Hodgkin's disease in childhood. © 1999 Cancer Research Campaign


*British Journal of Cancer* (1999) **80**(5/6), 808–814
© 1999 Cancer Research Campaign
Article no. bjoc.1998.0425

### Thyroid nodular disease after radiotherapy to the neck for childhood Hodgkin's disease

**EA Shafford[1], JE Kingston[1], JC Healy[2], JAW Webb[2], PN Plowman[3] and RH Reznek[2]**

Departments of [1]Paediatric Oncology, [2]Diagnostic Imaging and [3]Radiotherapy, St Bartholomew's Hospital, London UK

**Summary** Patients who receive radiotherapy to the neck are at risk of developing thyroid dysfunction. This prospective study of patients whose treatment for Hodgkin's disease in childhood included radiotherapy to the neck aimed to investigate the incidence and natural history of thyroid dysfunction and the morphological changes of the gland demonstrated on ultrasound. Forty-seven patients were investigated by clinical examination, thyroid function tests and thyroid ultrasound. Only six patients had a clinically detectable abnormality, but 64% had abnormal thyroid function tests. All patients had an abnormal thyroid ultrasound scan and 42% had at least one focal abnormality. A significant association was found between the presence of a focal lesion on ultrasound and young age at radiotherapy, longer follow-up and the length of time that the thyroid-stimulating hormone (TSH) level had been elevated. During follow-up, 65% of patients not on thyroxine developed new focal abnormalities. The longest time interval between radiotherapy and an increase in TSH level was 94 months, and from radiotherapy to the appearance of a focal abnormality on thyroid ultrasound was over 18 years. Three patients were found to have a thyroid carcinoma. These findings indicate the importance of long-term follow-up for patients treated by neck irradiation for Hodgkin's disease in childhood.

**Keywords:** childhood Hodgkin's; thyroid disease; neck irradiation

Patients who receive radiotherapy to the neck are at increased risk of developing thyroid dysfunction – usually biochemical hypothyroidism, but occasionally clinical hypothyroidism or thyrotoxicosis (Green et al, 1980; Kaplan et al, 1983; Constantine et al, 1984; Hancock et al, 1991; Peerboom et al, 1992). They are also more likely to develop both benign and malignant nodules of the thyroid gland (Stewart et al, 1989; Soberman et al, 1991). In 1991, we started a prospective study of the thyroid gland in patients whose treatment for Hodgkin's disease in childhood had included radiotherapy to the neck. The aim of the study was to gather information on the natural history of thyroid nodular disease following neck radiotherapy for childhood Hodgkin's disease and, using this information, to devise a protocol for the appropriate follow-up of these patients.

### METHODS

The thyroid gland of 47 patients who had received radiotherapy to the neck for Hodgkin's disease during childhood was prospectively investigated by: (i) clinical examination, (ii) thyroid function tests, and (iii) thyroid ultrasound scan. These investigations were repeated at approximately yearly intervals. Thyroid function tests undertaken prior to entry into the study were also reviewed.

For patients with a raised thyroid-stimulating hormone (TSH) level, the time interval between radiotherapy and the first elevated TSH level, and also the length of time that the TSH level remained raised, were calculated. Only patients whose first TSH level was recorded within 2 years of radiotherapy and who had levels measured regularly at no more than 2-yearly intervals were included in this calculation. When the TSH was elevated and remained elevated for 12 months, the patient was started on thyroxine.

All the ultrasound scans were carried out by one of three radiologists (JCH, RHR, JAWW). Focal abnormalities were identified and, on follow-up scans, any change to previously noted abnormalities or new focal lesions was recorded. Patients with a focal abnormality of 0.5 cm or greater were further investigated by radionuclide imaging and/or fine-needle aspiration biopsy (FNA). A small number of patients underwent thyroid surgery when the nature of the abnormality noted on the ultrasound scan remained in doubt.

### Analysis

Statistical analysis was done using the Minitab software. Patients with, and without, a focal abnormality on the first thyroid ultrasound scan were compared looking at: gender, age at radiotherapy, length of follow-up since radiotherapy, presence of a raised TSH level and the length of time the TSH level remained elevated. The $\chi^2$ test was used for comparison of proportions and the Mann–Whitney test for continuous variables.

### RESULTS

A total of 47 patients with Hodgkin's disease diagnosed between 1973 and 1989 were included in this study. Their characteristics are shown in Table 1. All patients had received radiotherapy to the neck, 14 to the neck alone and 33 to the neck and mediastinum. The median dose to the neck was 35 Gy (range 2250–4000 cGy).

*Received 17 June 1998*
*Revised 31 October 1998*
*Accepted 20 November 1998*

*Correspondence to:* EA Shafford, Department of Paediatric Oncology, The Royal London Hospital, Whitechapel, London E1 1BB, UK

*Present address: Department of Radiology, Chelsea and Westminster Hospital, 396 Fulham Road, London SW10 9NH, UK

### Clinical abnormalities

At the time of their initial scan, six patients had abnormal clinical findings. Five patients had palpable abnormalities of the thyroid gland or neck and were all more than 10 years from radiotherapy (Table 2). The remaining patient had signs of thyrotoxicosis 4 years from radiotherapy.

### Thyroid function tests

Thirty patients had abnormal thyroid function tests, either at the time of the first scan or documented at some time previously, with all but one patient, who had thyrotoxicosis, having a raised TSH level. The median time interval from radiotherapy to the first elevated TSH level was 27 months, range 2–94 months. Only two patients had a low level of thyroxine. Thirteen patients were on thyroxine at the time of their first scan and four were subsequently started on thyroxine because of a persistently elevated TSH level. In 12 patients, the TSH level had returned to normal spontaneously prior to the start of the study, over a period of time ranging from 9 to 100 months, median 33 months.

### Ultrasound scans

All patients had an abnormal thyroid ultrasound scan. Forty-five patients (95%) had diffuse atrophy whilst two had a goitre. Twenty patients (42%) had a focal abnormality. The majority (15) of these patients had multiple focal abnormalities, but five patients had a single focal abnormality. The size of the focal lesion was also variable and in five patients the lesion was > 1 cm. More detail of the sonographic abnormalities which can occur after radiotherapy to the neck for childhood Hodgkin's disease is given in the paper by Healy et al (1996).

Characteristics of patients with a focal abnormality compared to those without a focal abnormality are shown in Table 3. Using the $\chi^2$ test a significant association was demonstrated between focal abnormality and younger age at radiotherapy ($P < 0.02$) and longer follow-up ($P < 0.01$). There was also an association between focal abnormality and the length of time that the TSH level remained elevated ($P < 0.09$). However, no relationship was demonstrated between the presence of a focal abnormality and gender, or a raised TSH level.

Table 4 shows the frequency of abnormal thyroid function tests and thyroid nodules on ultrasound related to lymphangiography and dose of radiotherapy. These differences were not significant.

Ten patients had an ultrasound scan within 5 years of radiotherapy. Only two patients developed a focal abnormality within this time period. Both of these patients had evidence of autoimmune thyroid dysfunction with thyroid auto antibodies, one patient having thyrotoxicosis and the other hypothyroidism.

### Relationship of thyroxine therapy to the development of thyroid nodular disease

In order to obtain information on the natural history of thyroid nodular disease after neck radiotherapy for childhood Hodgkin's disease, patients were divided into two groups: those not on thyroxine and those taking thyroxine at the point of entry into the study. Four patients were started on thyroxine during the course of the study and were therefore censored from the first group at that point.

### Group 1 – patients not on thyroxine

Thirty-four patients were not on thyroxine at the time of the initial scan. Median follow-up from radiotherapy to first thyroid ultrasound scan was 13 years 5 months (range 6–21 years). Five patients are excluded from the following data. Three of these patients had surgery because of the findings on the initial scan, and two patients were started on thyroxine because of a persistently elevated TSH level.

Twenty-nine patients, nine of whom had a focal abnormality on the initial scan, had between two and five further scans over a period of time ranging from 3 to 6 years, median 5 years 3 months.

In four patients the multiple focal abnormalities noted on the initial scan remained unchanged throughout the study. Two of the patients had lesions less than 0.5 cm, one of whom had never had a raised TSH level, whilst the other had been treated for thyrotoxicosis. The other two patients had lesions greater than 1 cm in size. Both had previously had elevated TSH levels which had returned to normal spontaneously (after 78 months in one patient) prior to the commencement of the study. TSH levels subsequently remained normal.

Five patients, whose initial thyroid ultrasound scan was abnormal, had an increase in size or number of the focal abnormalities. Two of these patients are known to have had elevated TSH levels, whilst the other three had periods between 3 and 5 years when no levels were measured but all recorded levels were normal.

Six patients with no focal lesion on the first scan did not develop any focal abnormality during the course of the study. Five of these patients had never had a raised TSH level recorded. The sixth patient had had one elevated TSH level recorded but no more levels were measured until 7 years later when the TSH was normal.

Fourteen patients, whose initial scan was normal, developed focal abnormalities during the course of the study at a median of 11 years 10 months (range 6–18 years) from radiotherapy. The focal abnormality varied in size from > 1 cm (two patients), 0.6–1 cm (two patients) to 0.5 cm or less (ten patients). Three patients developed solitary lesions, the remainder developed between two and five nodules. Eight of these 14 patients had a raised TSH level, four during the study period and four at some time previously. Another patient had an abnormal thyrotrophin-releasing hormone (TRH) test one year prior to and at the commencement of the study. Only two of the remaining five patients are known never to have had a period of over stimulation of the thyroid gland by TSH. Doubt remains about the other three patients because, although all the recorded TSH levels are within the normal range, there were long periods of time between some of the measurements.

Apart from one cystic lesion, no focal lesion present on the initial scan disappeared during the course of the study. In this patient other focal abnormalities were also present in the thyroid gland and these persisted.

The percentage of patients with a focal lesion at 10 years and 15 years from radiotherapy was calculated. To do this several assumptions were made. During the course of the study although we demonstrated an increase in the number of focal lesions, in no patient did the number of focal lesions decrease, therefore any patient who had a focal lesion but had not reached 10 years follow-up from radiotherapy was included in the calculations for both time periods. Any patient whose initial scan showed a focal lesion but whose scan was done between 10 and 15 years from radiotherapy was only included in the calculation of the percentage with focal

© Cancer Research Campaign 1999

**Table 1** Patient characteristics

| | |
|---|---|
| Total number of patients | 47 (31 Male + 16 Female) |
| Stage at diagnosis | |
| Stage 1 | 15 patients |
| Stage 2 | 18 patients |
| Stage 3 | 12 patients |
| Stage 4 | 2 patients |
| No of patients with B symptoms | 15 |
| Median age at radiotherapy | 12 years 4 months (range 4 years 7 months to 16 years 7 months) |
| Median interval from radiotherapy to first scan | 10 years 4 months (range 9 months to 17 years 9 months) |
| Median length of follow up | 14 years 5 months (range 6 years 2 months to 22 years 11 months) |
| Median dose of radiotherapy to neck | 35 Gy (range 22.5–40 Gy) |
| No. of patients who had chemotherapy | 36 |
| No. of patients who had a lymphangiogram | 27 |

lesions at 15 years. If the inital scan was more than 10 years from radiotherapy, but did not show a focal lesion, this patient was included in the denominator for this calculation. The estimated percentage of patients not on thyroxine developing a focal lesion by 10 years from radiotherapy was 39% and by 15 years was 71%.

### Group 2 – patients on thyroxine

Thirteen patients were on thyroxine at the start of the study, median length of follow-up from radiotherapy to the first ultrasound scan for this group of patients was 10 years 3 months (range 4–16 years). One patient subsequently relapsed and died. The remaining 12 patients, eight of whom had a focal abnormality on the initial scan had between four and six scans over a period of time, ranging from 4 to 6 years. It is difficult to get a clear picture of the behaviour of thyroid nodules after suppression of TSH by thyroxine, as the thyroid glands of most of these patients had been subjected to long periods of over-stimulation by TSH (median 41 months, range 4–84 months) before thyroxine was commenced. Also, in the majority of patients, even after starting thyroxine the TSH level was not fully suppressed. However, in one patient who was fully suppressed (TSH level < 1 mu $l^{-1}$), two focal lesions < 0.5 cm present on the initial scan were no longer apparent on a repeat scan 1 year later and did not recur during 4 years of subsequent follow-up. A patient who had surgery for a solitary 1 cm ‘cold’ nodule present on the initial scan who had previously had a raised TSH level for 88 months did not develop any new nodules in the remaining thyroid lobe over a period of 4 years during which time the TSH level was fully suppressed. Two other patients showed a decrease in the size and number of focal abnormalities during the course of the study. In the remaining four patients who had focal abnormalities on the initial scan, there was an increase in either the size or the number of nodules during the course of the study. None of these patients were adequately suppressed.

Four patients who did not have a focal abnormality on the initial scan developed focal lesions. In one patient this was a transient solitary < 0.5 cm nodule which appeared when thyroxine was discontinued for 6 months, but disappeared when thyroxine was restarted. Two patients developed multiple nodules of 0.6–1 cm in size. Neither of these patients were adequately suppressed, one because of non-compliance and the other because the dose of thyroxine was initially too low. The fourth patient developed a solitary 0.5 cm nodule 12 years after commencing thyroxine. He had only been adequately suppressed for the previous 5 years because of non-compliance. This patient also had thyroid autoantibodies.

### Radionuclide scans

Twenty-six patients out of the total of 47 were further investigated with radionuclide imaging (RNI) because of lesions of 0.5 cm or greater in size on the ultrasound scan. Fourteen were reported as showing no focal defect, five a goitre, one a functioning ‘hot’ nodule, and six a ‘cold’ nodule. RNI was not as sensitive as ultrasound in identifying thyroid nodules. The smallest nodule identified on RNI measured 1.3 cm compared to 0.2 cm on ultrasound scan. No new nodules were detected by RNI.

### Fine-needle aspiration

In 11 patients, FNA biopsy was performed under local anaesthetic and ultrasound guidance, because of continuing concern about the exact nature of the lesion. All of these patients had multiple focal abnormalities with at least one lesion 1 cm or greater in size. A biopsy was performed because of an increase in size or an increase in the solid component of the focal lesion on serial scanning, and in one patient because of a grossly abnormal thyroid gland. In five patients, this procedure was non-contributory as only blood was obtained in the aspirate. In the other six patients, the aspirate contained small groups of follicular cells but no definite evidence of malignancy.

### Surgery

Ten patients underwent thyroid surgery, three of whom were subsequently found to have a thyroid carcinoma. One of these patients was found to have an isolated ‘cold’ nodule 13 years after radiotherapy to the neck at the age of 11 years. She had received 25 Gy in 15 fractions over 19 days. All recorded TSH levels were normal, so she was not on thyroxine prior to surgery. A needle biopsy was not performed. Histology of the thyroid gland showed

 *© Cancer Research Campaign 1999*

**Table 2** Abnormal clinical findings.

| | |
|---|---|
| Palpable thyroid nodule | 2 |
| Diffuse goitre | 2 |
| Lymph node anterior triangle | 1 |
| Signs of thyrotoxicosis | 1 |

the presence of a follicular variant of a papillary thyroid carcinoma. The adjacent thyroid parenchyma showed peripheral vacuolation of colloid as seen in TSH stimulation. The other two patients had surgery because of a grossly abnormal thyroid gland with multiple nodules, even though the FNA showed no evidence of malignancy. One was 20 years from radiotherapy to the neck given when he was 12 years old. He received 35 Gy in 20 fractions over 27 days. He had had a raised TSH for 18 months, 3 years post-radiotherapy, but the TSH level returned to normal spontaneously and so he had not been started on thyroxine. Metastatic papillary carcinoma was found in one of three cervical nodes removed at surgery. Two hypoechoic nodules, thought to be lymph nodes, behind the lower pole of the left lobe of the thyroid had been noted on ultrasound scan. No focus of carcinoma was found on multiple sectioning of the thyroid gland. The other patient was also 20 years from radiotherapy given when he was 10 years old. He received standard mantle field radiotherapy to a dose of 3000 cGy. All recorded TSH levels were normal, although there was a 5-year period, 12 months post-radiotherapy, when no TSH measurements were done. Histological examination showed thyroid follicles of varying size, with, in one area, a collection of poorly formed follicles with crowded nuclei, compatible with a microscopic papillary carcinoma of the thyroid. In addition to the patient mentioned above, two other patients with 'cold' nodules had surgery without prior biopsy.

Five other patients had surgery, all of whom had previously had a FNA biopsy. Four patients had an unsuccessful biopsy, and the fifth had surgery because of concern remaining about a grossly abnormal gland, even though the biopsy showed no evidence of malignancy.

In addition to the three patients with a carcinoma of the thyroid gland, three other patients had a total thyroidectomy, one had a subtotal thyroidectomy and the remaining three patients had a partial thyroidectomy. Two of these latter three patients had a solitary lesion. No further thyroid nodules have developed since surgery over 1 and 4 years follow-up. Both patients are on thyroxine and are fully suppressed. The third patient had nodules in the remaining thyroid lobe. These have remained unchanged over a period of 3 years. This patient is also on a suppressive dose of thyroxine. Histological examination of all the thyroid glands resected has shown typical radiation induced changes (Doniach et al, 1987; Carr and Livolsi, 1989).

### DISCUSSION

This study confirms the diversity of thyroid disease which may affect patients whose treatment for Hodgkin's disease in childhood included radiotherapy to the neck (Green et al, 1980; Kaplan et al, 1983; Constantine et al, 1984; Stewart et al, 1989; Hancock et al, 1991; Soberman et al, 1991; Peerboom et al, 1992). It also emphasizes the need for continuing follow-up of this group of patients. Although the median time from radiotherapy to the first elevated TSH level was 26 months some patients may not develop subclinical hypothyroidism until many years later. In this study the longest documented time interval from radiotherapy to an elevated TSH level was 7 years 10 months but other studies have noted an even longer time interval, of up to 18 years (Hancock et al, 1991). Fifty-seven per cent of patients developed subclinical hypothyroidism and an additional two patients (4%) also had low serum thyroxine levels These results are comparable to those reported by Peerboom et al (1992) but others have noted a considerable variation in the frequency of subclinical hypothyroidism (36–63%) (Green et al, 1980; Constantine et al, 1984).

In 12 of our patients, elevated TSH levels returned to normal spontaneously after a median of 33 months. This has also been noted in some other studies (Constantine et al, 1984; Hancock et al, 1991) and suggests that there may be a case for discontinuing thyroxine therapy, given to patients with a raised TSH level, after a period of time, and performing a TRH test to ascertain whether thyroxine is still necessary. It is difficult to know how long after the start of thyroxine treatment this stimulation test should be carried out. We performed TRH tests in two patients 10 years after radiotherapy who had been on thyroxine for 5 years. In one patient, the TSH level had been elevated for 14 months and, in the other patient, for 63 months prior to the commencement of thyroxine treatment. In both patients the TRH test was grossly abnormal and thyroxine therapy was restarted.

This study confirms the insensitivity of thyroid palpation in

**Table 3** Comparison of patients with and without focal abnormalities on initial thyroid ultrasound scan

| | Focal abnormality | No focal abnormality |
|---|---|---|
| Total no. of patients | 20 | 27 |
| Male:female | 11:9 | 20:7 |
| Median age at radiotherapy | 11 years 2 months | 13 years 8 months |
| Range | 4 years 7 months to 15 years 9 months | 5 years 3 months to 16 years 7 months |
| Median time since radiotherapy | 12 years 1 month | 8 years 10 months |
| Range | 3 years 11 months to 17 years 4 months | 8 months to 14 years 9 months |
| No. of patients with raised TSH | 14 (70%) | 14 (51%) |
| Median time from radiotherapy to first elevated TSH level | 30 months | 19 months |
| Range | 2 months to 51 months | 3 months to 94 months |
| Length of time TSH raised | 3 years 10 months | 2 years 2 months |
| Range | 4 months to 11 years | 7 months to 5 years 2 months |
| No. of patients on T4 | 8 (40%) | 5 (19%) |

*© Cancer Research Campaign 1999*

**Table 4** Association of raised TSH level and thyroid nodules on ultrasound with lymphangiography and dose of radiotherapy

| | | Normal TSH | Raised TSH |
|---|---|---|---|
| Lymphangiogram | Yes | 10 | 17 (62%) |
| | No | 7 | 12 (63%) |
| Radiotherapy ≤ 2500 cGy | | 5 | 6 (54%) |
| > 2500 cGy | | 12 | 23 (66%) |
| | | **No focal abnormality** | **Focal abnormality** |
| Lymphangiogram | Yes | 5 | 22 (81%) |
| | No | 4 | 16 (80%) |
| Radiotherapy ≤ 2500 cGy | | 3 | 8 (72%) |
| > 2500 cGy | | 8 | 28 (78%) |

detecting thyroid nodules (Soberman et al, 1991; Crom et al, 1997). Although thyroid ultrasound scans have been performed prospectively many of our patients had been off treatment for more than 10 years before the first scan was done so it is not possible to say when the abnormality first appeared. However, from the data collected in ten patients who had scans less than 5 years from radiotherapy it seems that focal abnormalities are unlikely to appear in this time period in the presence of normal thyroid function tests. The number of patients not on thyroxine who developed a focal lesion increased with increasing time from radiotherapy, 39% at 10 years and 71% at 15 years. Kaplan et al (1983) also noted increasing frequency of palpable thyroid abnormalities with increasing length of follow-up.

Prolonged over-stimulation of the thyroid gland by TSH seems to be an important factor in the development of focal abnormalities. Fourteen of 29 patients not on thyroxine developed a focal lesion, having previously had a normal scan, and an additional five patients had an increase in the size or number of focal lesions. Ten of these 19 patients had had a raised TSH level and one had an abnormal TRH test on two occasions. Only two of these patients are known to have always had a normal TSH level. In the remaining six patients, although all the TSH levels recorded were normal, there were long periods of time between some of these measurements.

The precise role of thyroxine in preventing these abnormalities is difficult to determine from this study as many patients experienced prolonged periods of over stimulation before being started on thyroxine and few patients have been adequately suppressed. However, resolution of focal lesions was seen only in the patients on thyroxine. Fogelfeld et al (1989), in a study of 511 patients who had surgery for benign thyroid nodules arising after radiotherapy to the neck for benign disease in childhood, demonstrated that treatment with thyroxine reduced the number of recurrences of benign nodules but did not affect the rate of thyroid cancer.

Whilst there would seem to be general agreement about treating patients with a raised TSH level after radiotherapy to the neck, with thyroxine to prevent over-stimulation of the thyroid gland, the optimum treatment of patients who have a TSH level in the normal range but an abnormal thyroid ultrasound scan is less clear. Our current practice is to treat such patients with thyroxine to suppress the production of TSH. It is too early to say whether this policy has led to a reduction in the number of patients with abnormal thyroid ultrasound scans but during the course of this study patients not on thyroxine continued to develop new focal lesions and only in the patients on thyroxine was any decrease in the number of focal lesions demonstrated.

In our study there was no difference in the frequency of thyroid dysfunction dependent on whether or not the patient had had a lymphangiogram at diagnosis. This is in agreement with the findings of Fleming et al (1985). However, some studies have shown an increase in thyroid dysfunction associated with lymphangiography (Kaplan et al, 1983), whereas others have shown a protective effect (Green et al, 1980). Neither did we demonstrate any relationship between lymphangiography and focal abnormalities in the thyroid gland. Now that lymphangiography has been superseded by computerized tomography (CT) scan as a staging procedure, any possible effect will be seen in a diminishing number of patients.

In our study less than a quarter of the patients were treated with doses of 2500 cGy or less, but our results are in accord with those of Constine et al (1984) that patients in the low-dose group have a lower incidence of subclinical hypothyroidism. This study did not demonstrate a relationship between the dose of radiation and development of thyroid nodules but the range of dose was small (2250–4000 cGy) compared to the study of Crom et al (1997) in which they demonstrated an increased relative risk of developing thyroid nodules with increasing dose of thyroid irradiation.

RNI does not have a role in the regular follow-up of these patients. The smallest nodule detected by RNI in this study was 1.3 cm in size, and only seven of 27 nodules of 0.5 cm or greater in size, previously identified on ultrasound, were seen on RNI. When the radionuclide scans were reviewed without knowledge of the result of the ultrasound, the isolated nodule in the patient subsequently shown to have a thyroid carcinoma was not identified.

FNA biopsy has been shown by others (Gharib and Goeilner, 1993) to be a useful technique in the management of patients with thyroid nodules. In our limited experience we did not find this to be the case. In five of 11 patients the biopsy was non-contributory. In our patients, however, nodules being aspirated seldom exceeded 1.0–1.5 cm, smaller than in those patients presenting with palpable thyroid masses. Given that thyroid carcinoma may present with multiple small foci, and that benign and malignant lesions may coexist, the value of a negative biopsy in this situation is questionable.

Ten patients had thyroid surgery, the majority of whom had either total or subtotal thyroidectomy. It has been shown by Fogelfeld et al (1989) that recurrence of thyroid nodules after surgical removal in patients irradiated in childhood for benign conditions is related to the amount of thyroid tissue remaining. In view of these findings and the continuing risk of malignant change in a previously irradiated thyroid gland, unilateral lobectomy in a patient with multiple focal abnormalities of the thyroid on

*© Cancer Research Campaign 1999*

ultrasound is of doubtful benefit as the patient will need to be on thyroxine and will also need to continue to have regular thyroid ultrasound scans.

The three observed cases of thyroid cancer may be compared with an expected number of about one in 400 (for ages 10–29 years) based on cancer registration rates for England and Wales (G Draper, personal communication). This excess is almost certainly exaggerated since the registration rates are based on clinically diagnosed symptomatic cases, whereas the three cases in the present study were discovered during a period of close surveillance, using ultrasound, and at the time of diagnosis all patients were symptom-free. However, even allowing for this, the observed incidence of three cases in 47 patients is vastly in excess of the expected incidence of one in 400.

### Follow-up

There is no consensus of opinion on the best way to follow-up patients whose treatment for malignant disease has included radiotherapy to the neck. Clinical examination can be insensitive, whilst thyroid ultrasound, because of its sensitivity, may result in the detection of nodules of uncertain clinical significance – the so-called incidentalomas (Ezzat et al, 1994; Tan and Gharib, 1997). However, it is known that patients treated with radiotherapy to the neck are at increased risk of developing benign and malignant thyroid nodules. The data on non-irradiated patients indicate that intra-thyroidal papillary carcinoma has an excellant prognosis when diagnosed on the basis of a clinically palpable abnormality (De Groot et al, 1990). However, we cannot necessarily extrapolate those findings to the post-irradiation situation. Thus, until we know the prognosis of post-irradiation intra-thyroidal papillary carcinoma we recommend that periodic ultrasound examination should be included in the follow-up of patients treated with neck radiotherapy.

We suggest the following as a suitable protocol for the follow up of all patients whose treatment for Hodgkin's disease included radiotherapy to the neck.

1. Clinical examination with careful palpation of the thyroid gland.
2. Measurement of TSH and thyroxine levels every 6 months for the first 5 years and then annually.
3. If TSH levels remain elevated for 1 year, institute treatment with thyroxine in a dose sufficient to suppress the TSH level to $< 1$ mu $l^{-1}$.
4. For patients on thyroxine, because of previously elevated TSH levels, and a normal thyroid ultrasound, consider stopping thyroxine after 5 years and monitoring thyroid function tests to determine whether continuing treatment with thyroxine is necessary.
5. The first thyroid ultrasound should be done 3 years from the end of radiotherapy and yearly thereafter.
6. Fine needle aspiration biopsy of the thyroid gland and/or surgery should be considered for lesions of 1 cm or greater, or for any lesion which increases markedly in size or changes in character, especially in a patient with normal TSH levels.

### CONCLUSIONS

Ultrasonography is the most sensitive method for detecting thyroid abnormalities but cannot distinguish between benign and malignant nodules (Garcia et al, 1992). It can, however, be used to document change both in size and character of focal lesions. FNA biopsy, in centres with the necessary expertise, or surgery should be considered for patients with both solitary and multiple focal abnormalities of the thyroid gland when there are lesions measuring 1 cm or greater, and in particular for patients whose nodules show a marked increase in size or change in character on serial scanning. Suppressive doses of thyroxine are indicated for patients with an elevated TSH. The use of thyroxine should be considered, probably within a trial set-up, for patients with normal TSH levels who develop focal nodules to ascertain whether or not thyroxine results in regression of nodules and prevents the development of new nodules.

Long-term follow-up of patients treated by neck irradiation during childhood is essential because of the long natural history of the development of thyroid dysfunction.

### ACKNOWLEDGEMENTS

We thank Dr JP Monson, Reader in Medicine, Department of Medicine and Endocrinology, St Bartholomew's Hospital for his helpful comments, and Mrs Susan Howarth for expert secretarial assistance.

### REFERENCES

Carr RF and Livolsi VA (1989) Morphologic changes in the thyroid after irradiation for Hodgkin's and non-Hodgkin's lymphoma. *Cancer* **64**: 825–829

Constine LS, Donaldson SS, McDougall IR, Cox RS, Link MP and Kaplan HS (1984) Thyroid dysfunction after radiotherapy in children with Hodgkin's disease. *Cancer* **53**: 878–883

Crom DB, Kaste SC, Tubergen DG, Greenwald CA, Sharp GB and Hudson MH (1997) Ultrasonography for thyroid screening after head and neck irradiation in childhood cancer survivors. *Med Ped Oncol* **28**: 15–21

De Groot LJ, Kaplan EL, McCormick M and Straus FH (1990) Natural history, treatment and course of papillary thyroid carcinoma. *J Clin Endocrinol Metab* **71**: 414–424

Doniach I, Kingston JE, Plowman PN and Malpas JS (1987) The association of post-radiation thyroid nodular disease with compensated hypothroidism. *Br J Radiol* **60**: 1223–1226

Ezzat S, Sarti DA, Cain DR and Braunstein GD (1994) Thyroid incidentalomas. *Arch Intern Med* **154**: 1838–1840

Fleming ID, Black TL, Thompson EI, Pratt C, Rao B and Hustu O (1985) Thyroid dysfunction and neoplasia in children receiving neck irradiation for cancer. *Cancer* **55**: 1190–1194

Fogelfeld L, Wiviott MBT, Shore-Freedman E, Blend M, Bekerman C, Pinsky S and Schneider AB (1989) Recurrence of thyroid nodules after surgical removal in patients irradiated in childhood for benign conditions. *N Engl J Med* **320**: 835–840

Garcia CJ, Daneman A, McHugh K, Chan H and Daneman D (1992) Sonography in thyroid carcinoma in children. *Br J Radiol* **65**: 977–982

Gharib H and Goellner JR (1993) Fine-needle aspiration biopsy of the thyroid: an appraisal. *Ann Intern Med* **118**: 282–289

Green DM, Brecher ML, Yakar D, Blumenson LE, Lindsay AN, Voorhess ML, MacGillivray M and Freeman AI (1980) Thyroid function in pediatric patients after neck irradiation for Hodgkin's disease. *Med Pediatr Oncol* **8**: 127–136

Hancock SL, Cox RS and McDougall IR (1991) Thyroid diseases after treatment of Hodgkin's disease. *N Engl J Med* **325**: 599–605

Healy JC, Shafford EA, Reznek RH, Webb JAW, Thomas JM, Bomanji JB and Kingston JE (1996) Sonographic abnormalities of the thyroid gland following radiotherapy in survivors of childhood Hodgkin's disease. *Br J Radiol* **69**: 617–623

Kaplan MM, Garnick MB, Gelber R, Li FP, Cassady JR, Sallan SE, Fine WE and Sack MJ (1983) Risk factors for thyroid abnormalities after neck irradiation for childhood cancer. *Am J Med* **74**: 272–280

Peerboom PF, Hassink EAM, Melkert R, De Wit L, Nooijen WJ and Bruning PF (1992) Thyroid function 10–18 years after mantle field irradiation for Hodgkin's disease. *Eur J Cancer* **28A**: 1716–1718

Soberman N, Leonidas JC, Cherrick I, Schiff R and Karayalcin G (1991) Sonographic abnormalities of the thyroid gland in longterm survivors of Hodgkin's disease. *Pediatr Radiol* **21**: 250–253

© Cancer Research Campaign 1999

Stewart RR, David CL, Eftekhari F, Reid HL, Fuller LM and Fornage BD (1989) Thyroid gland: US in patients with Hodgkin's disease treated with radiation therapy in childhood. *Radiol* **172**: 159–163

Tan GH and Gharib H (1997) Thyroid incidentalomas: management approaches to non-palpable nodules discovered incidentally on thyroid imaging. *Ann Intern Med* **126**: 226–231

*© Cancer Research Campaign 1999*
